# Use of a Modified STROOP Test to Assess Color Discrimination Deficit in Parkinson's Disease

**DOI:** 10.3389/fneur.2018.00765

**Published:** 2018-09-12

**Authors:** Rebekah G. Langston, Tuhin Virmani

**Affiliations:** ^1^College of Medicine, University of Arkansas for Medical Sciences, Little Rock, AR, United States; ^2^Department of Neurology, University of Arkansas for Medical Sciences, Little Rock, AR, United States

**Keywords:** Parkinson's disease, visual loss, diagnostic test assessment, early marker of disease, color vision dysfunction

## Abstract

**Objective:** To objectively measure color vision dysfunction in idiopathic Parkinson's disease (iPD) using an easily administered, essentially free, modified Stroop test.

**Methods:** Sixty-one iPD patients and 26 age-matched controls (HC) were enrolled after IRB approval and performed congruent (CST) and incongruent (IST) modified Stroop tests consisting of 40 words in 10 colors arranged in a 5 x 8 grid. The scorer was blinded to participant diagnosis. Errors on IST were defined as type 1 (written word reported rather than color) or type 2 (color reported different from the written word or its color).

**Results:** The iPD group and the control group completed testing with similar CST performance. On the IST, 75.4% of iPD patients had type 2 errors (*p* = 0.001, OR 4.907, 95%CI 1.838–13.097) compared to 38.5% HC, with a positive predictive value of 82%. The mean number of type 2 errors was also higher in the iPD group, even with MoCA scores as a covariate in the analysis. Type 1 errors were not significantly different between the groups. A univariate logistic regression model with age, gender, MoCA, normalized IST completion time and the presence/absence of type 2 errors also resulted in type 2 errors as the only significant factor in the equation (*p* = 0.026).

**Conclusions:** The modified Stroop test incorporated into the clinical evaluation of a patient may provide a quick and inexpensive objective measure of a non-motor feature of iPD, which could help in the clinical diagnosis of iPD in conjunction with the motor assessments currently used by neurologists.

## Introduction

Visual dysfunction has long been considered a non-motor symptom of idiopathic Parkinson's disease (iPD). The association between iPD and loss of color vision is well-established in the literature (1–3), and color discrimination deficit could potentially serve as an early marker of iPD (4–6). Although several underlying mechanisms have been proposed, the connection between iPD pathogenesis and decline in visual capabilities remains incompletely understood (7). It has been found that visual impairment correlates with cognitive decline in iPD patients (8); however, it is not clear if cognitive deficits result in poorer performance on visual assessments, or if changes in vision disable patients to an extent that affects performance on cognitive tests. Visual symptoms are present in non-demented iPD patients (8, 9), and may be due in part to retinal dopaminergic deficiency (10–12). With this in mind, further exploration of the color vision dysfunction experienced by iPD patients is warranted.

Deficiencies in color discrimination have primarily been measured using the Farnsworth-Munsell 100 Hue test, the abbreviated Farnsworth panel D15 test, or the Lanthony desaturated D15 test in previous studies. Here we propose utilizing the Stroop effect (13) to design a simple, rapid, and essentially free method of assessing disrupted color vision that could be incorporated into the clinical evaluation of an iPD patient.

## Methods

Subjects were prospectively enrolled in this study after obtaining written informed consent following approval from the University of Arkansas for Medical Sciences Institutional Review Board (UAMS IRB #203234) and in compliance with the guidelines in the Declaration of Helsinki for research involving human subjects. Included subjects were aged 18–90 with a diagnosis of idiopathic Parkinson's disease based on UK brain bank criteria. Subjects with a Montreal Cognitive Assessment Score (MoCA) <10, inability to walk or with more than 1 fall/day, on antidopaminergic agents in the year prior to enrollment, or with inability to complete the evaluations in english were excluded.

All subjects underwent a complete Unified Parkinson's Disease Rating Scale (UPDRS) assessment, Hoehn and Yahr Staging of Parkinson's disease (H&Y score), and a cognitive screening using the MoCA (Table 1). The Stroop test (ST) was modified for this study and consisted of 40 colored words arranged in a 5 x 8 grid. For the congruent Stroop test (CST; Supplementary Figure 1), each box contained the name of a color that was printed in the same color ink as the name. Participants were asked to state the color of the word in each box not “what the word said.” The time to complete the test and the number of errors reported were noted. This was followed by the incongruent Stroop test (IST; Supplementary Figure 2), in which the arrangement of words was the same, but each box contained the name of a color printed in a different color ink from the name. Participants were again asked to state the color of the word in each box not “what the word said.” Time taken to complete the tasks was recorded, in addition to correct and incorrect responses.

**Table 1:** Analysis of the indicated parameters for all subjects.

	**Control (*n* = 26)**	**iPD (*n* = 61)**	***P*-value**	**Odds ratio (95% CI)**
**DEMOGRAPHICS**				
Age (years)	62.8 ± 7.7	66.3 ± 8.4	0.075^#^	
Gender (%female)	61.5%	47.5%	0.232^*^	0.566 (0.222–1.445)
MoCA (max 30)	27.4 ± 1.7	25.4 ± 3.4	0.010^+^	
UPDRS Motor score (max 108)	0.9 ± 1.2	14.4 ± 1.2	<0.001^+^	
UPDRS Total score (max 176)	2.6 ± 2.2	28.4 ± 11.0	<0.001^#^	
H&Y score	–	2.1 ± 0.7	–	
FOG-Q score	0.08 ± 0.39	6.5 ± 5.5	<0.001^+^	
Motor duration (years)	–	8.2 ± 7.7	–	
**CONGRUENT STROOP (CST)**				
Time to complete (s)	30.5 ± 11.1	31.4 ± 10.3	0.575^+^	
Any error	3.8% (*n* = 1)	4.9% (*n* = 3)	0.827^*^	1.293 (0.128–13.043)
**INCONGRUENT STROOP (IST)**				
Time to complete (s)	62.2 ± 17.4	87.0 ± 59.7	0.012^+^	
Time normalized to CST	2.2 ± 0.8	2.8 ± 1.5	0.077^+^	
Type 1 error	38.5% (*n* = 10)	49.2% (*n* = 30)	0.358^*^	1.548 (0.607–3.948)
No. of errors	1.8 ± 1.1 (*n* = 10)	4.6 ± 5.5 (*n* = 30)	0.043^+^	
Type 2 error	38.5% (*n* = 10)	75.4% (n = 46)	0.001^*^	4.907 (1.838–13.097)
No. of errors	2.4 ± 1.4 (*n* = 10)	4.1 ± 2.5 (*n* = 46)	0.041^+^	
PrP error	23.1% (*n* = 6)	54.1% (*n* = 33)	0.008^*^	3.929 (1.386–11.138)
No. of errors	1.3 ± 0.5 (*n* = 6)	2.8 ± 1.2 (*n* = 33)	0.012^+^	
Any error	53.8% (*n* = 14)	80.3% (*n* = 49)	0.011^*^	3.500 (1.292–9.481)
No. of errors	3.0 ± 2.5 (*n* = 14)	6.7 ± 5.5 (*n* = 49)	0.005^+^	
Any self-corrected	42.3% (*n* = 11)	63.9% (*n* = 39)	0.062^*^	2.417 (0.947–6.171)
No. self-corrected	1.5 ± 0.7 (*n* = 11)	2.2 ± 1.3 (*n* = 39)	0.092^+^	

For each type of error measured on the modified Stroop test, the percent of total subjects in a group that made that type of error is shown in order to indicate presence/absence of an error type within each group. Mean number of errors made by those subjects that did make a given type of error is shown for IST data. All values are reported as mean ± standard deviation. Statistical tests used:

# *One-way ANOVA*;

+ *Mann-Whitney U-test*;

* *Chi-square test*.

In the case of incorrect responses on the IST, the color reported was noted on the assessment sheet by the examiner (TV). Subsequently, blinded to the diagnosis of the subject, the scorer (RL) quantified for each IST the number of incorrect responses in the following categories: (1) word read out instead of actual color reported (e.g., the word “red” printed in blue ink reported as red; type 1 error) or (2) color reported different from the color of word or the written word (e.g., the word “red” printed in blue ink reported as orange; type 2 error). Any color named by a participant that was neither the written word nor its color was considered a type 2 error.

Statistical analysis was performed using SPSS version 24 (IBM). Normality was assessed using the Schapiro-Wilk test. The one-way ANOVA was used for analysis of parametric data and the Mann-Whitney U-test for non-parametric data. The Chi-Square test was used for comparisons between categorical variables. Correlation was assessed using the Pearson's correlation coefficient. An analysis of variance using MoCA scores and/or gender as covariates was performed to determine the effect of cognition and gender on type 2 errors. A univariate logistic regression model was also constructed with age, gender, MoCA scores, normalized IST completion time and presence/absence of type 2 errors as variables.

## Results

A total of 87 subjects were enrolled; 26 healthy controls and 61 patients with idiopathic Parkinson's disease. Mean age and gender were not significantly different between the groups (Table 1). The control group had higher MoCA scores (*p* = 0.010), lower UPDRS motor and total scores (motor *p* < 0.001; total *p* < 0.001), and lower FOG-Q scores (*p* < 0.001) than the iPD group. Performance on the CST was not significantly different between the two groups based upon completion time and number of subjects with errors (Table 1).

On the IST, significantly more subjects with iPD reported type 2 errors (*p* = 0.001, OR 4.907, 95%CI 1.838–13.097), but not type 1 errors (*p* = 0.358, OR 1.548, 95%CI 0.607–3.948; Table 1). Making one or more type 2 errors corresponded with a positive predictive value of 82% for a diagnosis of iPD. Of the subjects who made type 2 errors, the mean number of colors reported incorrectly was also significantly higher in the iPD group (Table 1). Age was correlated with the absolute number of type 2 errors made in the control group (*p* = 0.038), but not in the iPD group (*p* = 0.080) (Supplementary Table 1). A common error was the reporting of “Pink” words as “Purple” (PrP) with 54% of iPD patients making this error at least once compared to only 23% of controls (*p* = 0.008, OR 3.929, 95%CI 1.386–11.138), with a higher mean number of errors in the iPD group (Table 1). We normalized the time to complete the IST by the time taken to complete the CST (IST time/CST time) to eliminate any difference based on reading speed or reading comprehension speed, and found no significant difference between the groups.

The number of type 2 errors was negatively correlated with MoCA scores in the iPD group (*r* = −0.343, *p* = 0.007) but not in the control group (*r* = −0.151, *p* = 0.462) (Supplementary Table 1). To address the possibility that the inability to discriminate colors was associated primarily with the degree of cognitive impairment, we performed an analysis of variance using the MoCA score as a covariate with the two groups (control and iPD) as the fixed variable, and the presence/absence of type 2 errors as the dependent variable. Even with the MoCA score as a covariate, there was a significant difference in type 2 errors between the groups (*p* = 0.007). Gender differences in visual perception were also found in our PD group, with almost 90% of males with PD showing type 2 errors while 59% of females showed type 2 errors (*p* = 0.04, Chi-square). Using gender as a covariate as above, there was still a significant difference in type 2 errors between the groups (*p* = 0.002). Additionally, with both MoCA scores and gender as covariates together type 2 errors were still significantly different between the groups (*p* = 0.012).

To further examine the relationship of other relevant factors to disease status, we also performed a univariate logistic regression with age, gender, MoCA, normalized IST completion time and the presence/absence of type 2 errors. Using this model, only presence/absence of type 2 errors was significantly associated with disease status (*p* = 0.026), while age (*p* = 0.313), gender (*p* = 0.705), MoCA score (*p* = 0.058), and normalized IST completion time (*p* = 0.525) were not significant variables in the final equation.

While the mean MoCA scores were lower in the iPD group compared to controls (Table 1), there was no significant difference in scores between iPD subjects who had type 2 errors compared to those that did not (Table 2). Additionally, motor UPDRS and total UPDRS scores, disease duration, and Hoehn and Yahr rating scale scores were similar between the two groups of iPD patients suggesting that disease severity did not contribute to the impairment in color discrimination using our modified Stroop test.

**Table 2:** iPD subjects dichotomized by the presence or absence of Type 2 errors.

	**Type 2 errors absent (*n* = 15)**	**Type 2 errors present (*n* = 46)**	***P*-value**
Age (years)	64.5 ± 9.1	66.9 ± 8.1	0.694^#^
CST Time to complete	28.9 ± 8.2	32.2 ± 10.8	0.262^+^
IST Time to complete	59.8 ± 19.1	95.9 ± 65.7	0.002^+^
Normalized time	2.6 ± 1.2	3.1 ± 1.8	0.018^+^
MoCA (max 30)	26.6 ± 2.6	25.0 ± 3.5	0.112^+^
UPDRS Motor score (max 108)	12.1 ± 5.6	15.2 ± 7.6	0.185^+^
UPDRS Total score (max 176)	24.9 ± 10.8	29.5 ± 10.9	0.358^#^
Motor duration (years)	8.7 ± 6.7	8.1 ± 6.2	0.880^+^
Hoehn and Yahr Score	2.0 ± 0.8	2.2 ± 0.6	0.365^+^

All values are reported as mean ± standard deviation. Statistical tests used:

# *One-way ANOVA*;

+ *Mann-Whitney U-test*.

## Discussion

Previous studies have reported progressive color discrimination deficits in iPD patients (1, 2, 14, 15) that are not age-dependent (16). The present study also showed color vision dysfunction in iPD patients, with the iPD group making significantly more type 2 errors, but not significantly more type 1 errors, compared to controls. A secondary finding of our study is that “Pink” words were commonly reported as “Purple” on the IST, with this particular error occurring significantly more often in the iPD group. On the CST, completed by all participants before the IST, subjects were presented the same words and colors and given the same instructions as with the IST, but did not make the same “Pink” reported as “Purple” errors. Age-matched controls also did not make the same mistake as often, suggesting this was a perception rather than a naming error. Perception of color is an abstract concept and therefore difficult to concretely assess. Should the results of our study be reproducible in a larger patient population, the modified STROOP may be useful in the investigation of color vision deficits as an early sign of iPD.

While the Stroop effect does not appear to elucidate differences that are highly specific or highly sensitive for a diagnosis of iPD, it may be helpful in the initial clinical evaluation of iPD patients as well as in clarifying the connection between iPD and visual deficits. We do not expect the modified Stroop test results to be confounded by patient age using presence/absence of type 2 error as the measured outcome because number of type 2 errors was not correlated with age in our iPD sample population, and age was not a significant factor in a univariate logistic regression model. Although number of type 2 errors was negatively correlated with MoCA score, and a greater proportion of males had a type 2 error on exam, the presence/absence of type 2 error was still significantly different between iPD patients and controls when MoCA scores and/or gender were used as covariate. Cognitive impairment may therefore influence, but is unlikely to be the primary driver of, performance on the modified Stroop test. As patients with other diseases of aging such as Alzheimer's disease (AD) also have visual perception deficits (17), comparing an AD populations' performance on our modified STROOP will be a future goal.

Diagnostic accuracy of idiopathic Parkinson's disease and other parkinsonism syndromes has remained problematic, as definitive diagnosis is only possible at autopsy. In clinicopathological comparison studies of the diagnosis of parkinsonism syndromes, it has been found that 1 in 4 clinical iPD diagnoses made by general neurologists was incorrect (18, 19). Movement disorders specialists have increased diagnostic accuracy, but misdiagnosis still is common, especially early in the course of the disease (20). With a positive predictive value of 82%, making a type 2 error on the IST could significantly help in making a clinical diagnosis of iPD along with other clinical features currently taken into account when evaluating a patient. Employing the Stroop effect in the clinical setting could be a fast and effective adjunctive test of a non-motor feature of iPD to lend support to the movement disorders examination in the early stages of iPD.

## Author contributions

TV study design, acquisition, statistical analysis and interpretation of data. RL data analysis and interpretation.

### Conflict of interest statement

The authors declare that the research was conducted in the absence of any commercial or financial relationships that could be construed as a potential conflict of interest. The reviewer SH and handling Editor declared their shared affiliation.
