# Supplementary figures and images for: Use of a Modified STROOP Test to Assess Color Discrimination Deficit in Parkinson's Disease
Source: Front Neurol. 2018 Sep 12;9:765. doi: 10.3389/fneur.2018.00765 (PMC6143680; doi:10.3389/fneur.2018.00765)

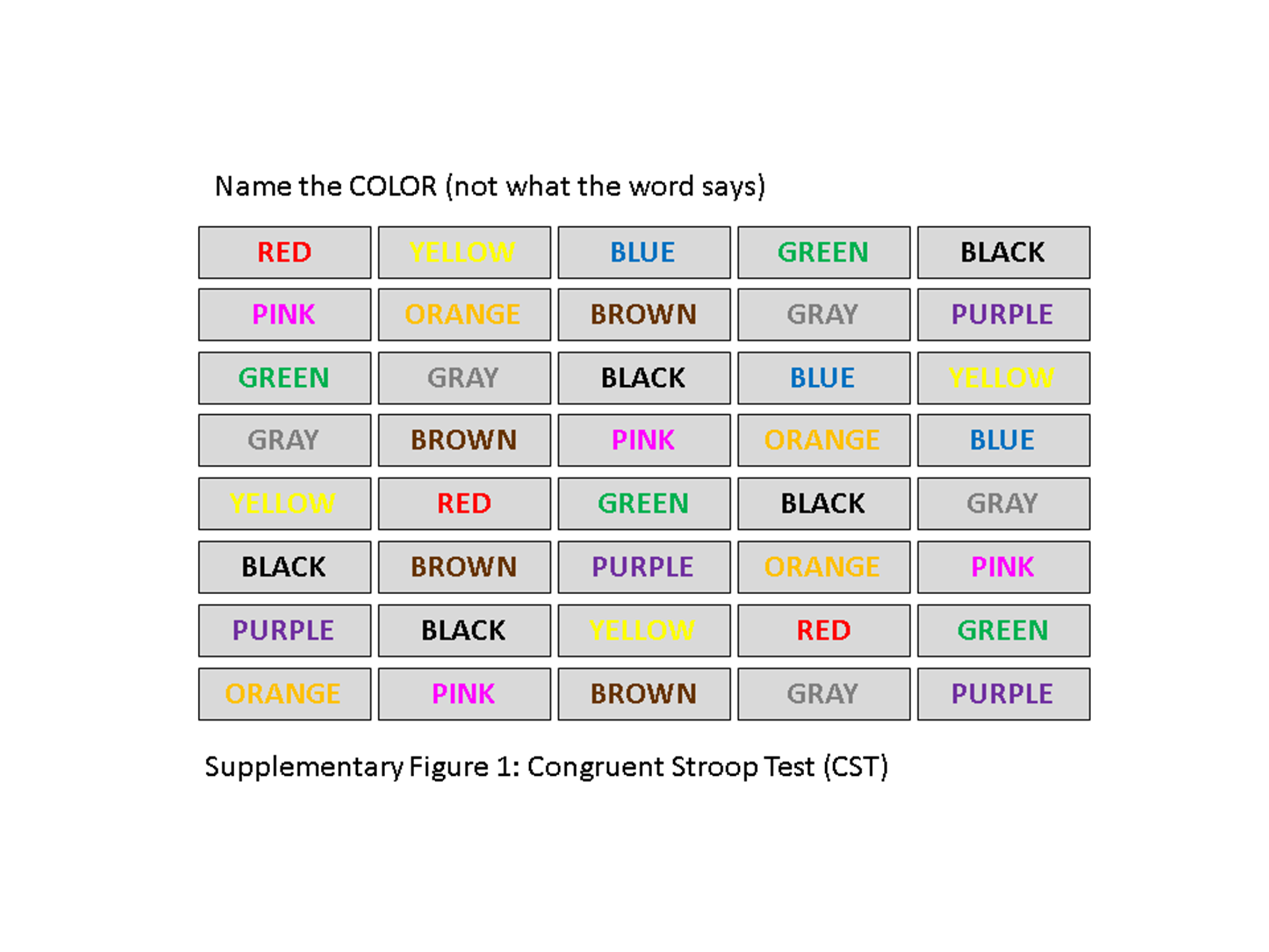

Supplement: Supplementary file 1 [file Image_1.tif]

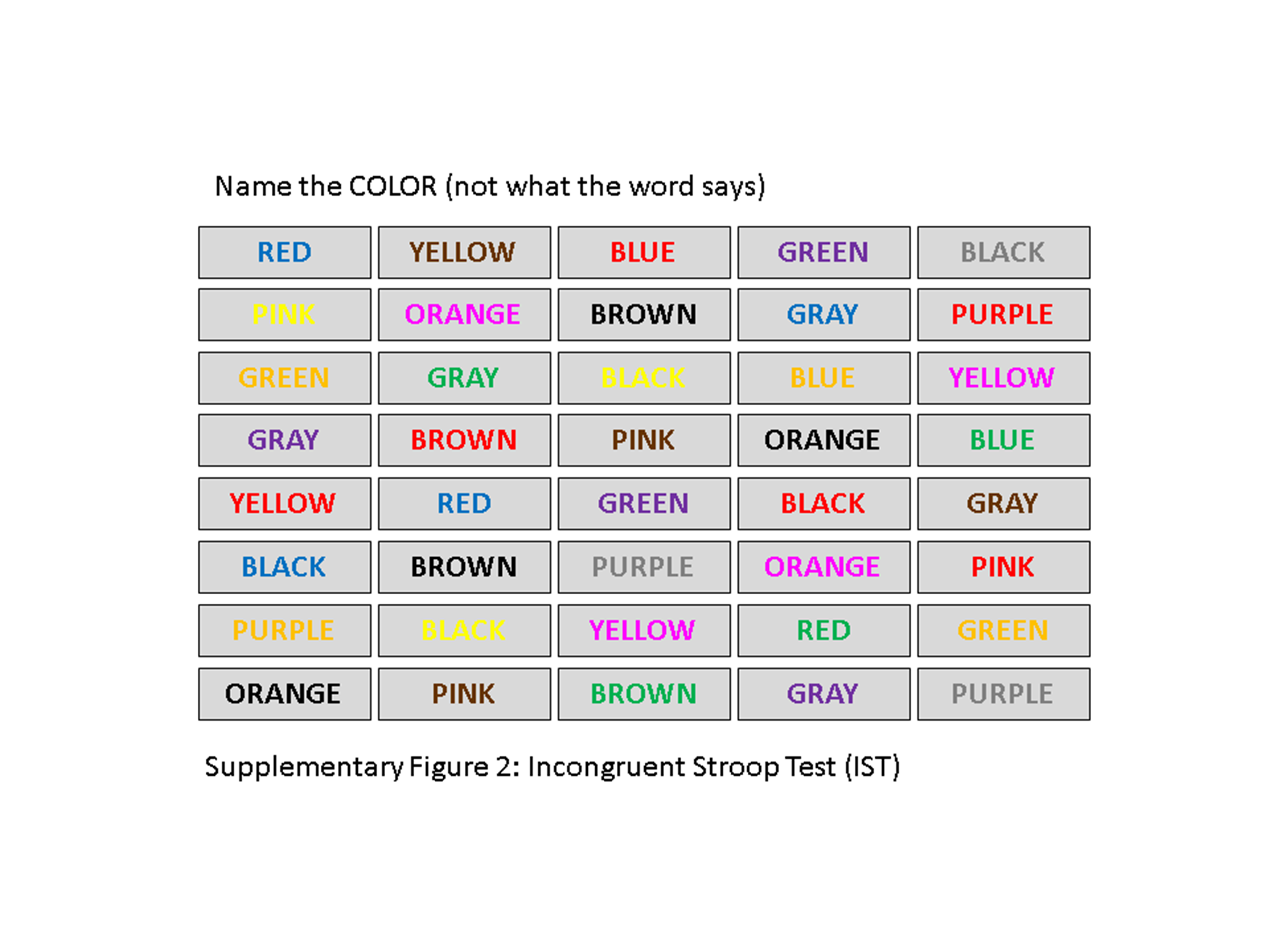

Supplement: Supplementary file 2 [file Image_2.tif]
