# Supplementary material for: Use of a Modified STROOP Test to Assess Color Discrimination Deficit in Parkinson's Disease
Source: Front Neurol. 2018 Sep 12;9:765. doi: 10.3389/fneur.2018.00765 (PMC6143680; doi:10.3389/fneur.2018.00765)
Supplement: Supplementary file 3 [file Table_1.DOCX]

Supplementary Table 1. Correlations between indicated variables and the absolute number of type 2 errors, based on Pearson’s correlation coefficient calculations.

|  | Control (n=26) | | iPD (n=61) | |
| --- | --- | --- | --- | --- |
|  | Pearson’s coefficient | P value | Pearson’s coefficient | P value |
| Age | 0.410 | 0.038 | 0.226 | 0.080 |
| H&Y |  |  | -0.029 | 0.825 |
| MoCA | -0.151 | 0.462 | -0.343 | 0.007 |
| UPDRS Motor score | 0.169 | 0.408 | 0.087 | 0.504 |
| UPDRS Total score | -0.143 | 0.485 | 0.104 | 0.424 |
| Motor duration |  |  | -0.076 | 0.561 |
| FOG-Q | -0.131 | 0.524 | -0.010 | 0.937 |
| IST Time | 0.689 | .002 | 0.343 | 0.007 |
| Normalized time (IST/CST) | 0.695 | <0.001 | 0.287 | 0.025 |
